# Inspiratory and Lower-Limb Strength Importance in Mountain Ultramarathon Running. Sex Differences and Relationship with Performance

**DOI:** 10.3390/sports8100134

**Published:** 2020-10-14

**Authors:** Ignacio Martinez-Navarro, Antonio Montoya-Vieco, Eladio Collado, Bárbara Hernando, Carlos Hernando

**Affiliations:** 1 Physical Education and Sports Department, University of Valencia, 46010 Valencia, Spain; Antonio.Montoya@uv.es; 2 Sports Health Unit, Vithas 9 de Octubre Hospital, 46015 Valencia, Spain; 3 Faculty of Health Sciences, Jaume I University, 12071 Castellon, Spain; colladoe@uji.es; 4 Department of Medicine, Jaume I University, 12071 Castellon, Spain; hernandb@uji.es; 5 Sport Service, Jaume I University, 12071 Castellon, Spain; hernando@uji.es; 6 Department of Education and Specific Didactics, Jaume I University, 12071 Castellon, Spain

**Keywords:** isometric strength, ankle reactive strength, pulmonary function, ventilatory efficiency, ultraendurance

## Abstract

The study was aimed at comparing lower-limb strength and respiratory parameters between male and female athletes and their interaction with performance in a 107 km mountain ultramarathon. Forty seven runners (29 males and 18 females; mean ± SD age: 41 ± 5 years) were enrolled. Lower-limb strength assessment comprised a squat jump test, an ankle rebound test, and an isometric strength test. Respiratory assessment included pulmonary function testing and the measurement of maximal inspiratory pressure. Male athletes performed largely better in the squat jump (26 ± 4 vs. 21 ± 3 cm; *p* < 0.001; d = 1.48), while no sex differences were found in the other two lower-limb tests. Concerning the respiratory parameters, male athletes showed largely greater values in pulmonary expiratory variables: forced vital capacity (5.19 ± 0.68 vs. 3.65 ± 0.52 L; *p* < 0.001; d = 2.53), forced expiratory volume in 1 s (4.24 ± 0.54 vs. 2.97 ± 0.39 L; *p* < 0.001; d = 2.69), peak expiratory flow (9.9 ± 1.56 vs. 5.89 ± 1.39 L/min; *p* < 0.001; d = 2.77) and maximum voluntary ventilation in 12 s (171 ± 39 vs. 108 ± 23 L/min; *p* < 0.001; d = 1.93); while no sex differences were identified in maximal inspiratory pressure. Race time was associated with ankle rebound test performance (r = −0.390; *p* = 0.027), isometric strength test performance (r = −0.349; *p* = 0.049) and maximal inspiratory pressure (r = −0.544; *p* < 0.001). Consequently, it seems that athletes competing in mountain ultramarathons may benefit from improving lower-limb isometric strength, ankle reactive strength and inspiratory muscle strength. Nevertheless, further interventional studies are required to confirm these exploratory results. In addition, the fact that the magnitude of the sex difference for isometric strength was minor, as compared with the other strength tests, could represent one of the factors explaining why the performance gap between males and females is reduced in ultramarathons.

## 1. Introduction

Lower-limb strength is significantly reduced following a mountain ultramarathon (MUM) [1,2,3,4,5]. Indeed, it seems that premature fatigue of lower-limb muscles might be one of the primary reasons why in MUMs, unlike in road marathons, heart rate generally decreases in the second half of the race and speed decay is more pronounced [6,7]. A possible relationship between muscular strength and MUM performance is therefore expected, as has been described in most endurance-based sports [8]. Lazzer et al. [9] found that lower-limb maximal power during a countermovement jump (CMJ) was correlated with a pre-to-post race increase in energy cost of running following a 43 km uphill MUM. However, Balducci et al. [5] found no relationship between CMJ height and performance in a 75 km MUM. Instead, these authors showed that race time was associated with knee extensor isometric force. As well as this controversy, no studies regarding a possible relationship between strength-related capacities and MUM performance have been conducted so far on female athletes.

Similarly, previous research has showed that MUMs provoke a significant respiratory function decline [10,11]. In addition, ventilatory muscle endurance (i.e., MVV_12_, maximum voluntary ventilation in 12 s) has been linked to performance in a 330 km MUM [10] and its intra-competition changes have been shown to be significantly related to the variance in running speed during a 24 h flat ultramarathon [12]. In fact, these latter authors suggested that the decrease in ventilatory muscle endurance may constrain running speed during extremely long ultramarathons. There is a lack of investigations, however, concerning a possible association between inspiratory muscle strength and MUM performance. Inspiratory muscle training using linear workload devices is accessible to athletes [13] and it constitutes an effective tool to improve endurance-sports performance [13,14,15]. Overall, further studies are warranted to resolve whether the limiting factors in ultra-endurance events are similar to those described in shorter distances [16], as well as studies comparing performance factors in male and female athletes competing in ultramarathon races [17].

The aims of our study were, therefore, two-fold. Firstly, to compare lower-limb strength and respiratory parameters between male and female MUM athletes. Secondly, to assess possible associations of lower-limb strength and respiratory function parameters with performance in a 107 km MUM, and to explore whether those relationships varied between male and female athletes. Our hypotheses were the following: greater lower-limb isometric strength, ankle reactive strength, ventilatory muscle endurance and inspiratory strength could be significantly related with a faster race time, and sex differences may be lower in those variables that correlate larger with performance.

## 2. Materials and Methods

### 2.1. Participants

Forty-seven ultra-endurance athletes (29 males and 18 females; 41 ± 5 years; 22.8 ± 2.1 kg/m^2^) from the Penyagolosa Trails CSP race in 2019 were selected to take part in the study. The race track consisted of 107.4 km, starting at an altitude of 40 m and finishing at 1280 m above the sea level, with a total positive and negative elevation of 5604 and 4356 m, respectively. All participants signed a written consent to participate and were informed about the procedures and the aims of the study. They were also allowed to withdraw from the study at any moment. Demographic information, as well as training and competition history were collected using an online questionnaire, as previously reported [18,19]. The investigation was conducted according to the Declaration of Helsinki and approval for the project was obtained from the research Ethics Committee of the University Jaume I of Castellon (Expedient Number CD/007/2019). This study is enrolled in the ClinicalTrails.gov database, with the code number NCT03990259 (www.clinicaltrials.gov).

### 2.2. Lower-Limb Strength Assessment

Subjects were familiarized with procedures concerning strength assessment during an informative session prior to the investigation. The following tests were carried out: (1) a squat jump test to assess lower-limb explosive strength; (2) an ankle rebound test to assess ankle reactive strength; (3) an isometric maximal voluntary contraction (IMVC) in a half-squat position. Each test was performed twice and the best performance was retained for statistical analysis. A 90 s recovery was used between attempts. Testing was conducted within 2 to 4 weeks before the race.

In the squat jump test, participants were asked to jump as high as possible from a starting position with hips and knees flexed 80 degrees and hands stabilized on hips to avoid arm-swing [19]. Jump height (SJ_height_) was estimated by the flight time measured with a contact platform (Chronojump, Barcelona, Spain). In the ankle rebound test, participants were asked to perform 10 consecutive maximal jumps with no knee flexion and the intention of minimizing ground contact time. The ratio between flight and contact times (Leg Q_Index_), measured by a contact platform (Chronojump, Barcelona, Spain), was considered for analysis.

IMVC was measured with a force sensor (Chronojump, Barcelona, Spain) held on to a bar using a custom-adapted Smith machine (i.e., the bar was firmly anchored to impede any movement). Participants adopted a half-squat position (i.e., hips and knees flexed 80 degrees) and afterwards they were instructed to push (as if they were to perform a dynamic contraction) as fast as possible and maintain the maximal isometric exertion during 5 s. We opted for a half-squat position to evaluate IMVC as this multi-articular assessment was expected to be closer to the specific joint angles required during running, especially when compared to monoarticular testing [20]. The highest force produced was modeled using an inverse monoexponential function considering the speed at which maximal force was reached. IMVC was relativized by body weight.

### 2.3. Respiratory Assessment

Pulmonary function testing was conducted using an automated online system (Oxycon Pro system, Jaeger, Würzburg, Germany), while the participant was seated and wearing a nose-clamp, following the American Thoracic Society and European Respiratory Society guidelines for spirometry standardization [21]. A 1 min recovery between attempts was used in all respiratory assessments. All the tests were performed by the same experienced investigator to guarantee that maneuvers were carried out properly [22]. Forced vital capacity (FVC), forced expiratory volume in 1 s (FEV_1_), FEV_1_/FVC ratio and peak expiratory flow (PEF) were determined from the maximal flow volume loop (MFVL). Each participant performed three acceptable MFVL maneuvers that lasted at least 6 s each one. The spirometric maneuver with the highest sum of FVC and FEV_1_ was accepted.

On the other hand, maximal inspiratory pressure (MIP) was measured using a handheld electronic device (Powerbreathe K5, HaB International Ltd., UK) [23]. This test was carried out to evaluate volitional maximal inspiratory strength. Each participant performed three attempts and the best result that did not differ by more than 5% was considered for analysis. This value was relativized by body weight. Lastly, respiratory muscle endurance was assessed using the MVV_12_ maneuver. Athletes were asked to breathe as rapidly and deeply as possible for 12 s and the best result from two attempts was retained for statistical analysis. MVV testing was performed last to avoid participant fatigue.

### 2.4. Statistical Analysis

Statistical analyses were performed with the aid of the Statistical Package for the Social Sciences software (IBM SPSS Statistics for Windows, version 22.0, IBM Corp., Armonk, NY). Normality was verified conducting the Shapiro–Wilk test. Since all the variables were normally distributed (*p* > 0.05), possible sex differences in lower-limb strength variables (SJ, IMVC, Leg Q_Index_), respiratory variables (FVC, FEV_1_, FEV_1_/FVC ratio, PEF, MVV_12_) and training-related data collected in the questionnaire were compared using a Student’s t-test and chi-square test (categorical data). On the other hand, possible associations between race time and lower-limb strength variables and respiratory variables were assessed using Pearson correlations. This analysis was carried out for the whole sample and for the males and females sample sets. The following criteria were considered to evaluate the magnitude of the correlations: r ≤ 0.1, trivial; 0.1 < r ≤ 0.3, small; 0.3 < r ≤ 0.5, moderate; 0.5 < r ≤ 0.7, large; 0.7 < r ≤ 0.9, very large; and r > 0.9, almost perfect; while a Cohen’s D between 0.3–0.5 was considered small; between 0.5–0.8, moderate; and greater than 0.8, large [24]. The significance level was set at *p* < 0.05 and data are presented as means and standard deviations (±SD).

## 3. Results

Four participants did not start the race due to injury. From a starting sample of 43 athletes, 32 runners (19 men and 13 women) successfully completed the race. The finishers/starters ratio for the participants of our study (74.4%) was similar to the ratio when all race participants were considered (73.8%). Male athletes’ average finish time was 20 h 43 min ± 3 h 58 min, while females athletes’ average finish time was 22 h 20 min ± 2 h 24 min. Those average finish times represented 174% of men’s winning time and 157% of women’s winning time, respectively. They ranked from 13th to 395th place (of 397 finishers) in the male category, and from 7th to 32nd place (of 47 finishers) in the female category; so the sample was highly heterogeneous regarding its performance level.

Sex differences in training-related data, lower-limb and respiratory variables are outlined in Table 1 and Table 2. No significant sex differences were identified in training-related data. Regarding lower-limb strength variables, SJ was largely greater in male athletes, while no sex differences were found in Leg Q_Index_ and IMVC. Lastly, concerning respiratory parameters, FVC, FEV_1_, PEF and MVV_12_ were largely greater in male athletes, whereas no sex differences were identified in FEV_1_/FVC and MIP.

Results from correlational analysis are depicted in Table 3. Considering the whole sample, leg Q_Index_ and IMVC were inversely and moderately correlated with race time, while MIP was inversely and largely associated with race time. In the male sample set, only MIP was largely correlated with performance; while in the female sample set, only Leg Q_Index_ was largely associated with performance.

## 4. Discussion

The present study aimed at analyzing sex differences in lower-limb strength and respiratory function and assessing which of those variables were correlated with race time in a 107 km MUM. Our results showed that male athletes exhibited significantly better values of lower-limb explosive strength, as compared to women. However, no sex differences were identified in ankle reactive strength and lower-limb isometric strength (IMVC). At the same time, those latter variables were significantly correlated with performance (Leg Q_Index_ in the entire sample and female sample set; IMVC in the entire sample), while SJ was not correlated with race time. On the other hand, inspiratory pulmonary function variables (except for FEV_1_/FVC) were significantly better among male athletes, while sex difference in MIP did not reach statistical significance. Interestingly, MIP was the only respiratory variable associated with performance (in the entire sample and male sample set).

Balducci et al. [5] found that knee extensors isometric force was correlated with race time in a 75 km MUM. Our results thus reinforce the relationship between isometric strength and MUM performance. Indeed, isometric strength training performed at a long muscle length is highly recommended to improve strength at biomechanically disadvantaged joint positions (i.e., as the ones that mountain runners sustain during downhill sections of MUMs) [25]. Therefore, its importance regarding MUM performance could be related with muscle fatigue subsequent to downhill running [26,27]. Moreover, percentage sex difference in this strength-related capacity was the least in the array of strength variables analyzed (5.3%). In the same line, Temesi et al. [28] demonstrated that force loss in the knee extensors following a 110 km MUM was lower among women as compared with men. Collectively, lower muscle fatigability and minor difference in isometric strength (compared to other strength-related capacities) could be two of the factors that explain why the performance gap between males and females is reduced in ultramarathons compared to shorter endurance events [29,30,31,32].

On the other hand, unlike Balducci et al. [5], we found a significant correlation between ankle reactive strength and performance. Although we performed the same test, the reason for this discrepancy may lie in the fact that Kleg (the variable assessed in the abovementioned study) considers body mass whereas Leg Q_Index_ (the variable analyzed in the present study) does not. Overall, leg stiffness improvement has been correlated with an increase in running economy [33] and ankle reactivity has been associated with better downhill running performance [20]. Consequently, it appears reasonable that ankle reactive strength was associated with MUM performance. In addition, this relationship was stronger among female athletes, so training strategies aimed at improving this strength-related capacity seem especially pertinent among women. Lastly, our results corroborate a lack of relationship between lower-limb explosive strength and performance in uphill–downhill MUMs [5,20].

The absence of a significant relationship between MVV_12_ and race time in our study contrasts with previous investigations undertook in flat and mountain ultramarathons [10,12]. Male participants in our study showed better values in this variable compared to the samples previously analyzed, so it could be that a ceiling effect exists regarding positive transfer effects from MVV to MUM performance; this would explain why we failed to find a significant relationship between MVV_12_ and race time. Notwithstanding, further studies are required to clarify whether MVV is associated with MUM performance. On the other hand, the significant association between MIP and race time reinforce previous suggestions about the importance of inspiratory muscle function in ultramarathon running [11,34]. Inspiratory muscle fatigue has been demonstrated to impair locomotor muscle performance because a larger fraction of total cardiac output is required by the respiratory muscles and a sympathetic vasoconstrictor response to working skeletal muscle is triggered through a respiratory muscle metaboreflex [35,36]. At the same time, it has been proposed that inspiratory muscle strength is a critical determinant of the magnitude of inspiratory muscle fatigue during prolonged endurance exercise [34,35]. Therefore, although we cannot infer a causal relationship from a correlation, our results reinforce the pertinence of a specific inspiratory muscle training in MUM runners, as it has been advocated for other endurance sports (i.e., road running, rowing, cycling) [13,14,15]. This specific inspiratory muscle training could be performed with the aid of a pressure threshold device, either in isolation or integrated into core workouts [37] and stationary cycling [38]. It is usually recommended to start with a protocol of 30 resisted breaths, twice daily, at an intensity corresponding to the 50% of MIP [13,14,15].

Moreover, as respiratory muscles play an important role regarding trunk stability [37,39,40] and post-MUM reductions in static and dynamic postural control have been documented [41,42], a better muscle inspiratory capacity may minimize the risk of fall and improve ventilatory efficiency and running economy in the last segments of the races [10,11,34,43]. Indeed, regarding ventilatory efficiency, a recent study has observed that during downhill running, as compared with uphill running, the ventilation pattern becomes more superficial (greater respiratory frequency and lower tidal volume) [20]; so inspiratory muscle training could aid to improve the ventilatory pattern during the downhill sections of MUM.

Some limitations of the study should be acknowledged. On one hand, we failed to perform any lower-limb muscular endurance tests, so we could not know whether a possible relationship existed between race time and lower-limb muscular endurance. Further studies are needed to clarify the relative importance of muscular strength vs. muscular endurance regarding MUM performance. On the other hand, as maximal oxygen uptake was not considered in the analysis, we cannot discard that the relationship between inspiratory strength and performance could be mediated by maximal oxygen uptake. Lastly, the whole set of lower-limb strength tests could not be performed following the MUM or in the days following the race, so we could not compare how race effort affected each strength-related capacity and its rate of recovery.

## 5. Conclusions

The findings of the present study suggest that lower-limb isometric strength and ankle reactive strength are two main lower-limb strength-related capacities regarding MUM performance. Consequently, it seems that strength training and assessment in athletes competing in those races should focus on the above-mentioned strength-related capacities. Nevertheless, further interventional studies are required to confirm these exploratory results. In addition, minor sex difference in isometric strength (compared to other strength-related capacities) could be one of the factors that explain why the performance gap between males and females is reduced in MUMs as compared with shorter trail running races. Lastly, athletes and coaches are encouraged to include inspiratory muscle training exercises into their daily routine to improve MUM performance.

## Figures and Tables

**Table 1:** Sex differences in training-related data.

	Males (*n* = 19)	Females (*n* = 13)	% Difference (Men vs. Women)	*p*	ES
Number of Years Running	8 ± 2	8 ± 3	−0.5%	0.969	0.01
Number of Races > 100 km	2 ± 3	2 ± 4	−3.9%	0.936	0.03
Weekly Training days	5 ± 1	5 ± 1	−2.3%	0.798	0.10
Weekly Running Volume (km)	76 ± 25	61 ± 13	19.4%	0.065	0.71
Weekly Positive Elevation (m)	1868 ± 765	1631 ± 565	12.7%	0.348	0.35
Weekly Training Hours	10 ± 4	9 ± 5	10.0%	0.520	0.24
Strength Training (yes/no)	74%/26%	92%/8%	−25.3%	0.185	-

Abbreviations: Strength training, percentage of participants who performed at least one weekly lower-limb strength training in the previous 3 months; ES, Effect Size.

**Table 2:** Sex differences in lower-limb strength and respiratory variables.

	Males (*n* = 19)	Females (*n* = 13)	% Difference (Men vs. Women)	*p*	ES
Leg Q_Index_	1.89 ± 0.33	1.72 ± 0.22	9.0%	0.111	0.61
IMVC (N/kg)	10.9 ± 2.71	10.32 ± 4.45	5.3%	0.650	0.17
SJ_height_ (cm)	26 ± 4	21 ± 3	19.7%	<0.001	1.48
FVC (L)	5.19 ± 0.68	3.65 ± 0.52	29.5%	<0.001	2.53
FEV_1_ (L)	4.24 ± 0.54	2.97 ± 0.39	29.9%	<0.001	2.69
FEV_1_/FVC (%)	81.95 ± 5.68	81.28 ± 2.63	0.8%	0.697	0.15
PEF (L/min)	9.9 ± 1.56	5.89 ± 1.39	40.5%	<0.001	2.77
MVV_12_ (L/min)	171 ± 39	108 ± 23	36.9%	<0.001	1.93
MIP (cm H_2_O/kg)	1.61 ± 0.33	1.39 ± 0.32	13.8%	0.068	0.70

Abbreviations: Leg Q_Index_, ratio between flight and contact times in ankle rebound test; IMVC, isometric maximal voluntary contraction; SJ_height_, squat jump height; FVC, forced vital capacity; FEV_1_, forced expiratory volume in 1 s; PEF, Peak expiratory flow; MVV_12_, maximum voluntary ventilation in 12 s; MIP, maximal inspiratory pressure; ES, Effect Size.

**Table 3:** Results from correlational analysis.

	Correlation with Race Time (r/*p*)		
	All Sample (*n* = 32)	Men (*n* = 19)	Women (*n* = 13)
Leg Q_Index_	−0.390/0.027	−0.278/0.249	−0.607/0.028
IMVC (N/kg)	−0.349/0.049	−0.371/0.118	−0.398/0.178
SJ_height_ (cm)	−0.324/0.070	−0.224/0.357	−0.290/0.336
FVC (l)	−0.277/0.125	−0.109/0.657	−0.318/0.289
FEV_1_ (l)	−0.239/0.188	−0.027/0.912	−0.310/0.303
FEV_1_/FVC (%)	0.175/0.337	0.199/0.415	0.200/0.511
PEF (l/min)	−0.213/0.242	0.060/0.807	−0.336/0.261
MVV_12_ (l/min)	−0.234/0.197	−0.096/0.695	−0.140/0.648
MIP (cm H_2_O/kg)	−0.544/0.001	−0.576/0.010	−0.384/0.195

Abbreviations: Leg Q_Index_, ratio between flight and contact times in ankle rebound test; IMVC, isometric maximal voluntary contraction; SJ_height_, squat jump height; FVC, forced vital capacity; FEV_1_, forced expiratory volume in 1 s; PEF, Peak expiratory flow; MVV_12_, maximum voluntary ventilation in 12 s; MIP, maximal inspiratory pressure.
